# Salvage of bleeding renal allograft following biopsy, with suture technique: a case report

**DOI:** 10.1186/s13256-016-0870-2

**Published:** 2016-04-02

**Authors:** Aruna Prasanna, Ranga Migara Weerakkody, Eranga Sanjeewa Wijewickrama, Mohammed Rezni Nizam Cassim, Mandika Wijeyarathne

**Affiliations:** 1 Vascular and Transplant Division, University Surgical Unit, National Hospital of Sri Lanka, Regent Street, Colombo 10, Sri Lanka; 2 Renal Services, University Medical Unit, National Hospital of Sri Lanka, Regent Street, Colombo 10, Sri Lanka; 3 Department of Clinical Medicine, Faculty of Medicine, University of Colombo, Kynsey Road, Colombo 8, Sri Lanka; 4 Vascular and Transplant Division, Department of Surgery, Faculty of Medicine, University of Colombo, Kynsey Road, Colombo 8, Sri Lanka

**Keywords:** Renal biopsy, Arteriovenous fistula, Allograft, Bleeding

## Abstract

**Background:**

Percutaneous renal biopsy is a valuable procedure in the management of and prognostication for patients with renal disease. Complications, although rare, occur with renal biopsies. Arteriovenous fistulas and heavy bleeding are notable complications. In this report, we describe simple suturing of the biopsy tract for salvage of a graft destined for a nephrectomy due to a profusely bleeding arteriovenous fistula.

**Case presentation:**

A 20-year-old Sri Lankan man with end-stage renal disease due to steroid-resistant nephrotic syndrome underwent a renal transplant. He had poor urine output following the surgery, and a renal biopsy was performed to diagnose his renal pathology. He experienced poorly controlled postprocedural hypertension, and he had four episodes of gross hematuria that required blood transfusion. Coil embolization was delayed due to technical issues, and a graft nephrectomy was planned following the fourth episode of hematuria, which was the most severe. A Doppler scan revealed a slender, iatrogenic arteriovenous fistula corresponding to the biopsy tract, with very high flow rates. With knowledge of the anatomy of the fistula, we performed suturing of the tract to obliterate the fistula as a last resort to salvage the graft. The surgical procedure stopped the bleeding, and the patient made a full recovery with an excellent quality of life.

**Conclusions:**

In our patient, a renal transplant biopsy revealed acute tubular necrosis. The incidence and treatment of fistulas and differences in complication rates among native and graft kidney biopsies are discussed.

## Background

Percutaneous renal biopsy provides crucial evidence in the assessment and management of allograft dysfunction. It is an invasive procedure with complications, and most of them are related to bleeding. Sometimes the complications are severe enough to require a nephrectomy or to result in death. Hematuria, hematoma formation, arteriovenous fistulas (AVFs), pseudoaneurysm, and clot retention are the commonest complications related to bleeding [1–3]. Most of the bleeding episodes respond to conservative management, including bed rest, blood transfusions, and correction of clotting abnormalities. Troublesome bleeding requires active intervention, coil embolization, or nephrectomy. We report a case of a patient in whom suturing of a biopsy tract containing an AVF was used to stop the bleeding and eventually salvage the graft.

## Case presentation

A 20-year-old Sri Lankan man diagnosed with end-stage renal failure associated with childhood steroid-resistant nephrotic syndrome underwent a renal transplant. His altruistic donor had ABO and human leukocyte antigen compatibility within acceptable limits. The patient was not sensitized and had received massive doses of immunosuppressants, including steroids, cyclosporine, tacrolimus, azathioprine, mycophenolate, and cyclophosphamide. He had severe growth retardation with height (1.45 m) and weight (26 kg) well below the third percentile, lack of secondary sexual characteristics, and cushingoid features. He had severe hypertension causing end-organ damage, retinopathy, and left ventricular hypertrophy. Control of his blood pressure had been challenging before the transplant.

He received induction immunosuppression keeping with local induction protocol (intravenous basilixumab 20 mg, cyclosporine 20 mg/kg/day, mycophenolate mofetil 25 mg/kg/day, and intravenous methylprednisolone 1 g). His cold and warm ischemia times were 60 and 24 minutes, respectively. Following surgery, he was managed in a high-dependency unit for monitoring and support. Standard immunosuppressive therapy was commenced thereafter.

The patient had poor urine output and became anuric 4 h after the transplant. Ultrasound-guided renal biopsy was carried out by a trainee nephrologist on the second day after the transplant to assess graft failure. Before the patient’s biopsy, his hemoglobin level was 117 g/L, his platelet count was 178,000/μl, his creatinine level was 563 μmol/L, and his international normalized ratio (INR) was 1.1. We used real-time ultrasound to guide the biopsy and a Bard® 18-gauge spring-loaded disposable gun (Bard Biopsy Systems, Tempe, AZ, USA) to collect tissue. With two firings of the gun, we obtained two tissue cores. The patient’s blood pressure before the biopsy was 160/100 mmHg, but postbiopsy it rose to 220/120 mmHg. Immediately after the procedure, the patient complained of severe suprapubic pain, and gross hematuria with clots was noted. Urgent surgical review was sought, and bladder irrigation was started via a cystostomy. The patient’s volume depletion was treated with crystalloids and packed red blood cell transfusions. His blood pressure was controlled with intravenous nitroglycerin and labetalol. Within a couple of hours, we were able to control the patient’s bleeding. However, he had three further episodes of bleeding amounting to 500–750 ml per episode, which was massive in relation to his body weight. Twelve units of blood were transfused in total to control his hypovolemia over the course of 60 h. Fortunately, the patient did not develop massive transfusion syndrome. His highest INR was 1.3, his longest activated partial thromboplastin time was 39.4 seconds, and his lowest platelet count was 130,000/μl. We did not obtain a thromboelastogram before the surgery, and desmopressin was not used preoperatively, in keeping with the local protocol.

**Fig. 1:**
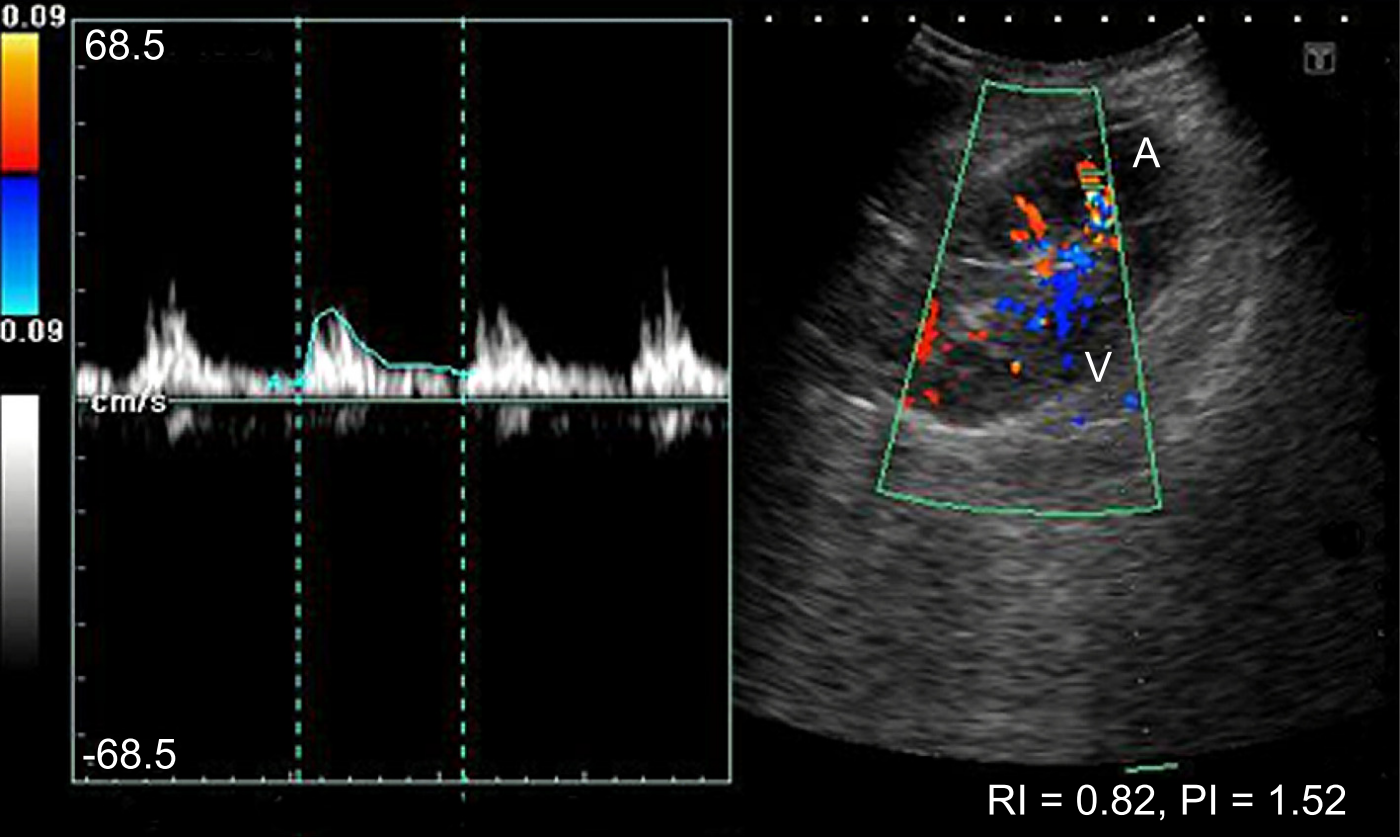
Doppler sonographic image of the allograft. The superior pole contains a lesion with turbulent flow. The feeding artery (*A*) and the draining vein (*V*) are visible in *red* and *blue*, respectively. The lesion shows a mixed arterial and venous flow with a resistivity index of 0.82. The rest of the kidney showed no diastolic flow, and the resistivity index was 1.0

**Fig. 2:**
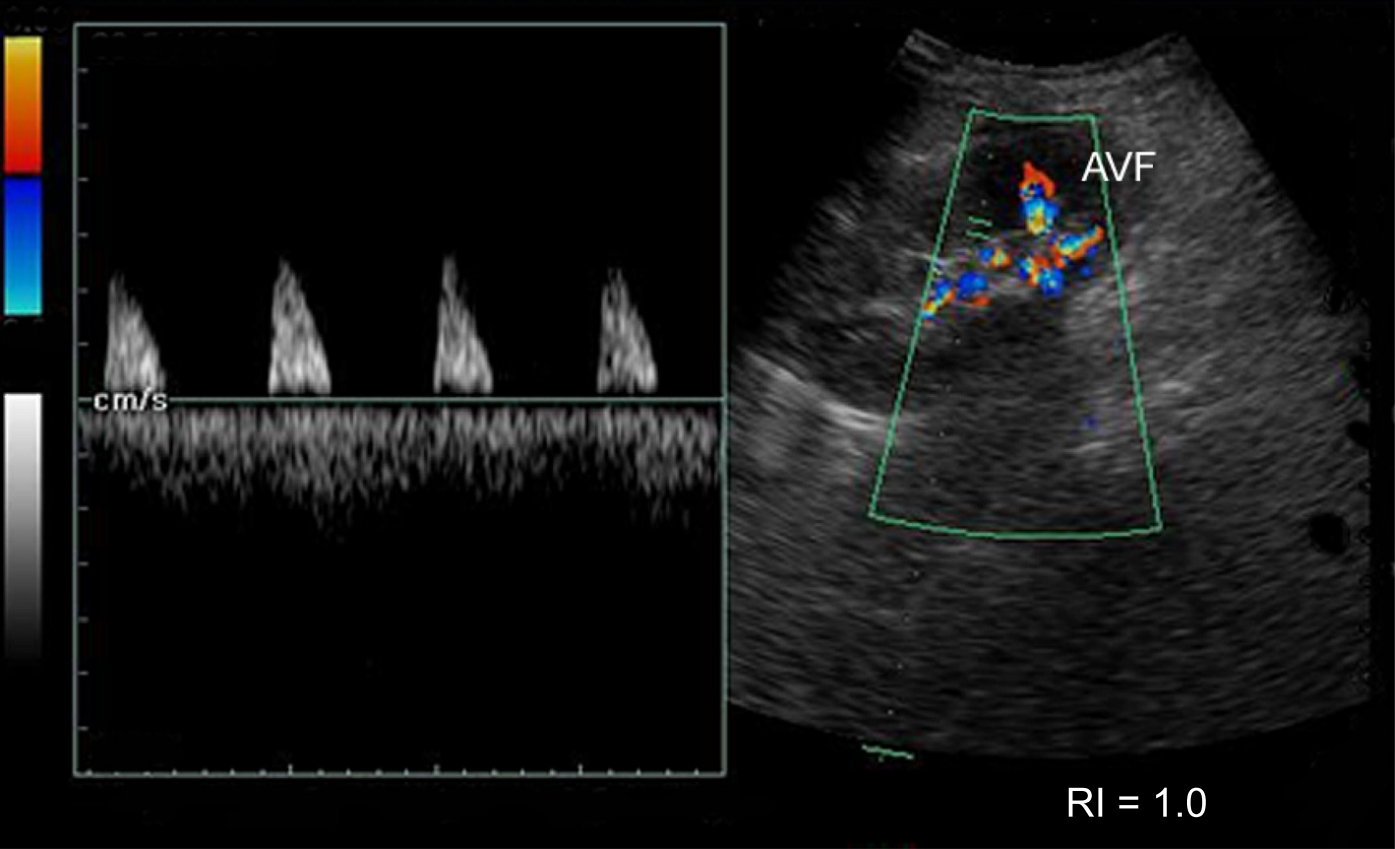
Doppler sonographic image of the allograft. The flow of the middle pole is assessed in the figure. The arteriovenous fistula (*AVF*) is visible in the superior pole. Monophasic systolic flow and absent diastolic flow are prominent features, leading to a radiological diagnosis of acute rejection

**Fig. 3:**
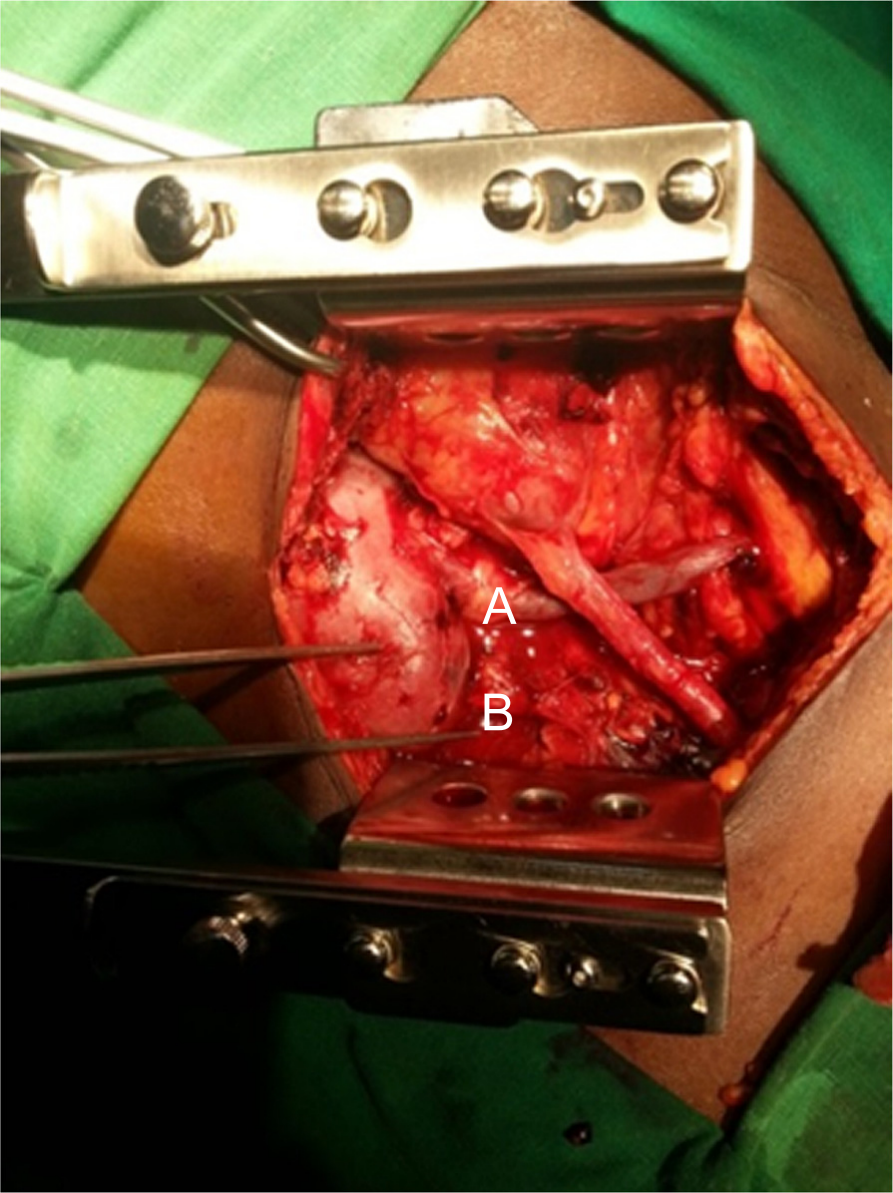
The surface of the kidney has two biopsy tracts with a common entrance (catch inserted to elaborate) and two separate exits (*A* and *B*). Tract *A* was bleeding profusely

A duplex ultrasound scan showed the presence of an iatrogenic, slender AVF (2–3 mm in width and 25 mm long) in close proximity to the biopsy tract (Fig. 1), as well as a small, perinephric hematoma (not shown). The flow of the AVF was as great as 150 ml/minute and resulted in poor diastolic flow in the rest of the kidney. The pulsatile flow and high resistivity index of 1.0 presented a clinical picture similar to that of acute rejection (Fig. 2). We planned to selectively embolize the feeding vessels of the AVF; however, the interventional radiology department of the National Hospital of Sri Lanka was experiencing technical difficulties, and selective embolization was possible only on the following morning. The patient developed another torrential bleed of 1250 ml in the evening on day 2, and an emergency graft nephrectomy appeared to be the only way to stop his bleeding. His skin was opened through the existing surgical incision, and we observed a profusely bleeding biopsy tract (Fig. 3). A minor perinephric hematoma of about 100 ml was observed. The patient’s bladder was distended with clots and close to rupture. We were well aware of the anatomy of the AVF, which was slender. We decided to control the bleeding using local pressure (that is, by suturing the tract).

**Fig. 4:**
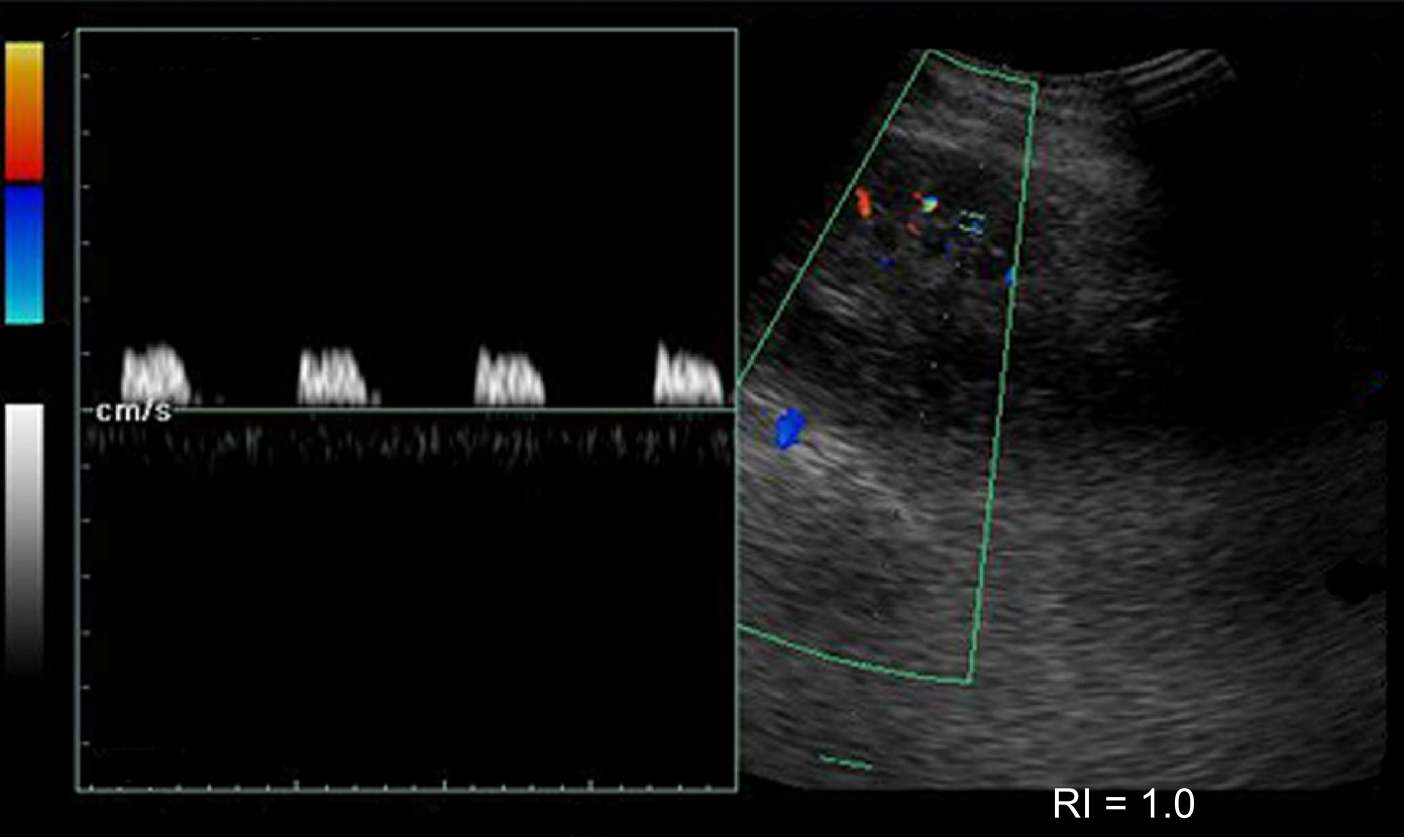
Doppler sonogram obtained immediately following suturing of the biopsy tract. The flow pattern has changed to biphasic, but diastolic flow is still absent. Note the loss of peripheral circulation on the sonogram. The patient’s resistivity index was 1.0, and the radiological diagnosis was consistent with that of acute tubular necrosis

**Fig. 5:**
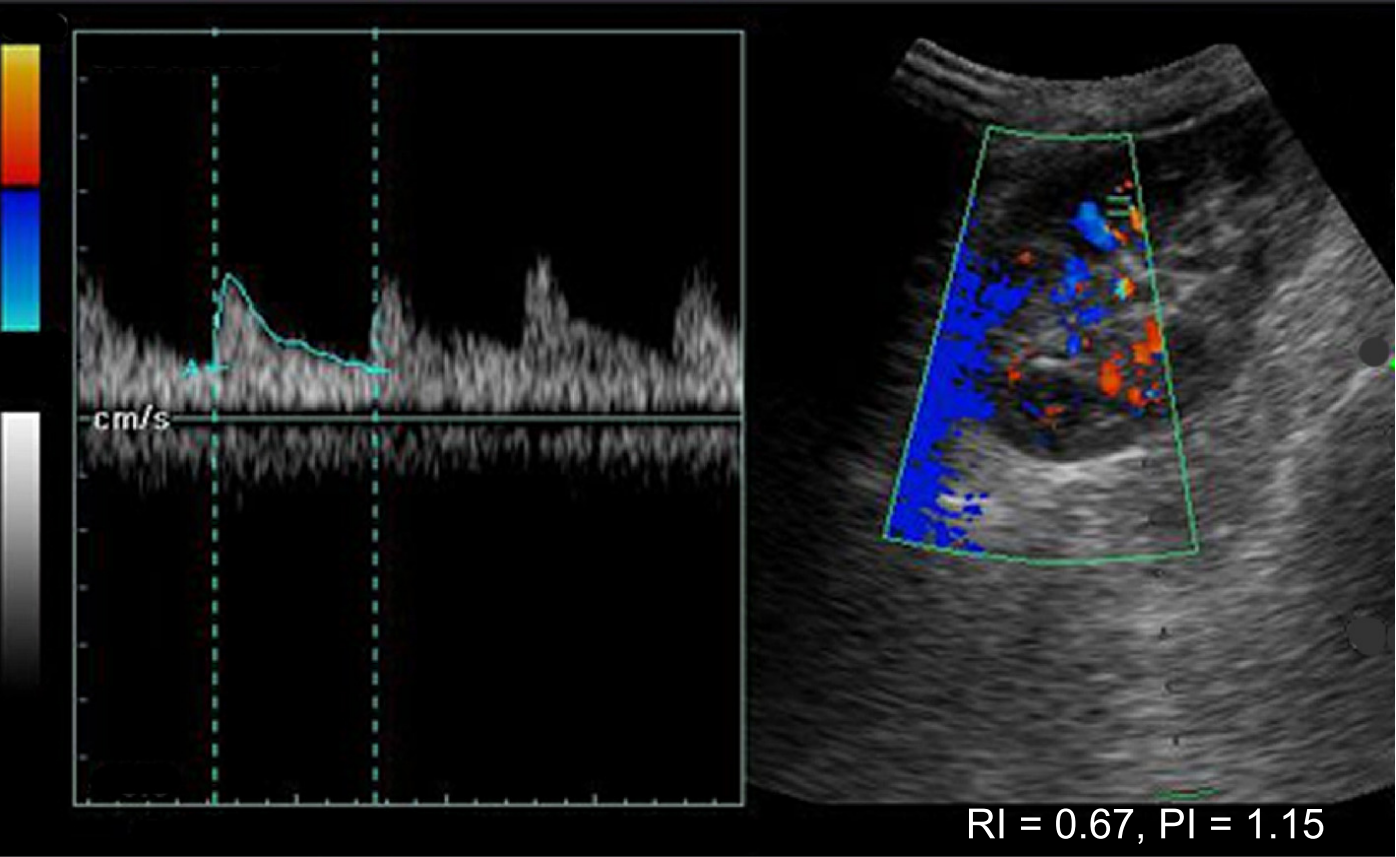
Doppler sonogram of the allograft 6 weeks later shows flow estimation in the upper pole where the arteriovenous fistula was identified. The scan does not show any turbulent flow or feeding vessels

**Fig. 6:**
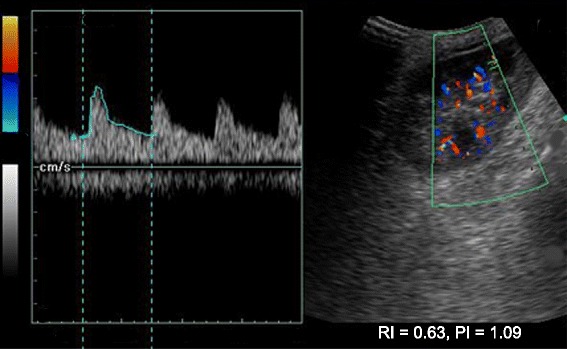
Doppler sonogram of the allograft 6 weeks later shows flow estimation in the lower pole. The patient’s resistivity indices are 0.63–0.67 and flow patterns are normal, which are features of a normal allograft

We used a #0 polyglactin 910 filament to suture the bleeding AVF with an encircling knot cutting through the renal parenchyma. The patient’s bleeding resolved, and pulsatile flow in the area surrounding the sutured segment (15 % of the surface area of the kidney) was absent. Suturing was hindered by edema of the surrounding parenchyma, as tight sutures tend to cut through the parenchyma. We evacuated clots via a cystostomy and inserted a suprapubic drain. The patient did not experience any further bleeding. A repeat duplex study showed obliteration of the AVF and change of the flow patterns (from monophasic to biphasic), suggestive of acute tubular necrosis (Fig. 4). A renal biopsy confirmed the radiological diagnosis, and the patient was recommenced on hemodialysis. His urine output improved by day 16 posttransplant, and his creatinine levels reached a baseline of 116 μmol/L by day 20. He was discharged on day 22, and at his 18-month follow-up he was doing well, with a creatinine level of 108 μmol/L. His height, secondary sexual characteristics, and quality of life had improved. A repeat duplex scan done 6 weeks after the biopsy showed no features of AVF and excellent perfusion of the graft (Figs. 5 and 6).

## Discussion

Renal biopsy is regarded as the gold standard for the diagnosis, prognosis, and management of patients with renal disease. The first renal biopsy dates back more than a century [4]. Advancements in the needle, as well as use of real-time ultrasonography, have gradually improved the technique and the yields of the biopsies [5]. The safety of the procedure has improved considerably, even in the hands of trainee physicians [5, 6]. However, renal biopsy can cause minor to serious complications. Bleeding manifestations include hematuria, hematoma, and profuse bleeding with a consequent need for blood transfusions, coil embolization, or surgical procedures such as nephrectomy; rarely, death can result [3]. Serious complications are rare when risk factors such as hypertension, bleeding, and clotting tests are within acceptable ranges [7, 8]. Our patient had severe hypertension periprocedurally, which is a well-known risk factor for bleeding. His platelet count and coagulation were within reference ranges. Hypertension is a risk factor for, as well as a consequence of, bleeding. Compression of the kidney by a subcapsular hematoma—commonly following a biopsy—is termed *Page kidney*, which is a recognized cause of secondary hypertension. The acute rise of our patient’s blood pressure could have been secondary to a Page allograft kidney. A small perirenal hematoma supports this possibility, though the majority of the bleeding was into the renal pelvis and then into the bladder. Classically, this has been described in native kidneys, but it has been reported in transplanted kidneys as well [9].

Needle biopsies of renal allografts are performed per protocol or for indicated biopsies. Our patient underwent the latter type. The overall reported incidence of bleeding in allograft biopsies varies greatly, ranging from 3 % to 16.5 %, and the reported incidence of symptomatic AVFs is about 0.4 % [3, 10, 11]. The complication rates of graft biopsies compared with native biopsies is a subject of debate. Researchers in some studies have reported lower complication rates in graft biopsies (28.9 % versus 19.5 %, *p* = 0.033) [12–14], while others have shown twice as many serious complications (hematoma, hemoperitoneum, and AVF) [10]. The management of complications is straightforward. Bed rest itself resolves most of the minor bleeding episodes, and only severe bleeding episodes require blood transfusion, radiological intervention, and nephrectomy.

In native renal biopsies, AVFs occur at rates of 1–18 % [15, 16]. Around 39 % of them are symptomatic. The majority (87 %) resolve spontaneously, and only 13 % require treatment [15]. Gross hematuria occurs in less than 10 % of patients with acquired AVF [17]. Our patient had torrential bleeding episodes, and we did not have access to interventional radiology facilities overnight. Coil embolization carries an excellent prognosis and should be regarded as the first line of therapy. The procedure we describe in this report was done as a last resort to salvage the kidney. To the best of our knowledge, this is the first reported case of suturing of a biopsy tract containing an AVF to salvage the graft.

## Conclusions

Exploratory surgery can effectively and promptly control bleeding and correct underlying anatomical problems. All efforts must be made to salvage the viable allograft.

## Consent

Written informed consent was obtained from the patient for publication of this case report and any accompanying images. A copy of the written consent is available for review by the Editor-in-Chief of this journal.

## Abbreviations

AVF: arteriovenous fistula
INR: international normalized ratio
